# Etheno adducts: from tRNA modifications to DNA adducts and back to miscoding ribonucleotides

**DOI:** 10.1186/s41021-021-00199-x

**Published:** 2021-06-16

**Authors:** F. Peter Guengerich, Pratibha P. Ghodke

**Affiliations:** Department of Biochemistry, Vanderbilt University School of Medicine, 638B Robinson Research Building, 2200 Pierce Avenue, Nashville, TN 37232-0146 USA

**Keywords:** Etheno modification, Bis-electrophiles, Chemical carcinogens, DNA adducts, RNA adducts, Mutagenesis, X-ray crystallography, Mass spectrometry, Enzyme kinetics

## Abstract

Etheno (and ethano) derivatives of nucleic acid bases have an extra 5-membered ring attached. These were first noted as wyosine bases in tRNAs. Some were fluorescent, and the development of etheno derivatives of adenosine, cytosine, and guanosine led to the synthesis of fluorescent analogs of ATP, NAD^+^, and other cofactors for use in biochemical studies. Early studies with the carcinogen vinyl chloride revealed that these modified bases were being formed in DNA and RNA and might be responsible for mutations and cancer. The etheno bases are also derived from other carcinogenic vinyl monomers. Further work showed that endogenous etheno DNA adducts were present in animals and humans and are derived from lipid peroxidation. The chemical mechanisms of etheno adduct formation involve reactions with *bis*-electrophiles generated by cytochrome P450 enzymes or lipid peroxidation, which have been established in isotopic labeling studies. The mechanisms by which etheno DNA adducts miscode have been studied with several DNA polymerases, aided by the X-ray crystal structures of these polymerases in mispairing situations and in extension beyond mispairs. Repair of etheno DNA adduct damage is done primarily by glycosylases and also by the direct action of dioxygenases. Some human DNA polymerases (η, κ) can insert bases opposite etheno adducts in DNA and RNA, and the reverse transcriptase activity may be of relevance with the RNA etheno adducts. Further questions involve the extent that the etheno adducts contribute to human cancer.

## Introduction

**Fig. 1:**
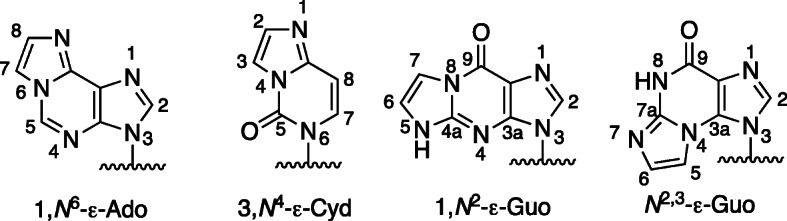
The four major etheno bases. Numbering systems are shown. The modified bases may be in DNA or RNA

**Fig. 2:**
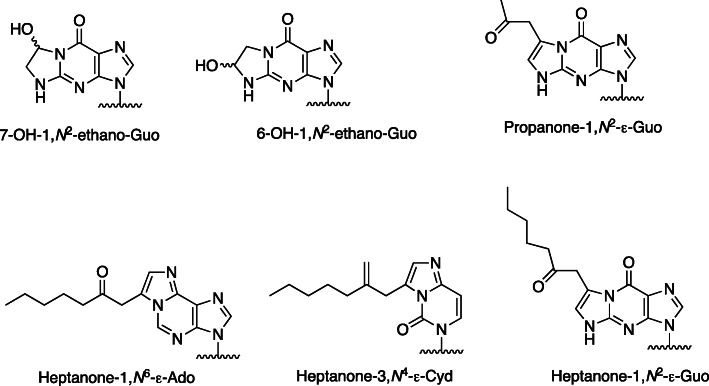
Other etheno derivatives reported in biological systems [1–4]

Etheno adducts are interesting for a number of reasons. One of us was first introduced to these in the late 1970s, and both of us continue to work with these today. The most important four etheno adducts, at least with regard to issues of mutagenesis and cancer, are shown in Fig. 1. Note the numbering systems, which differ from the purines and pyrimidines. Some additional 5-membered exocyclic ring compounds of relevance are shown in Fig. 2.

The history of etheno bases goes back to before either of us knew about them, to their discovery in tRNA. These are natural products. The history continues through organic synthesis and use in bioorganic chemistry and then their discovery as DNA adducts derived from work with chemical carcinogens. Detailed mechanisms of formation are discussed. Another important discovery was their presence as “endogenous” DNA adducts. The effects of the etheno adducts on DNA pairing have been investigated in detail, both in free oligonucleotides and within active sites of DNA polymerases. The etheno DNA adducts are repaired by specific enzymes. Finally, etheno adducts in RNA can be misread by DNA polymerases involved in reverse transcriptase activity.

## Y-bases in tRNA

**Fig. 3:**
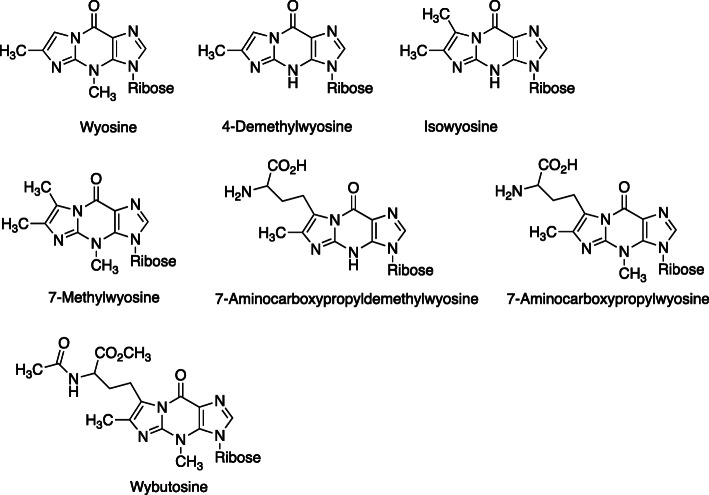
Wyosine and related tRNA bases containing etheno structures [12]

tRNAs have a number of unusual bases, which appear to be involved in maintaining the stem-loop structures needed. A fluorescent base was found in yeast tRNA [5–7], and structures were characterized [8–11]. These structures (Fig. 3) are derivatives of 1,*N*^2^-ε-Guo (Fig. 1). They have been found in tRNAs from animals, yeast, and archaebacteria but not eubacteria or in mitochondria or chloroplasts [12].

**Fig. 4:**
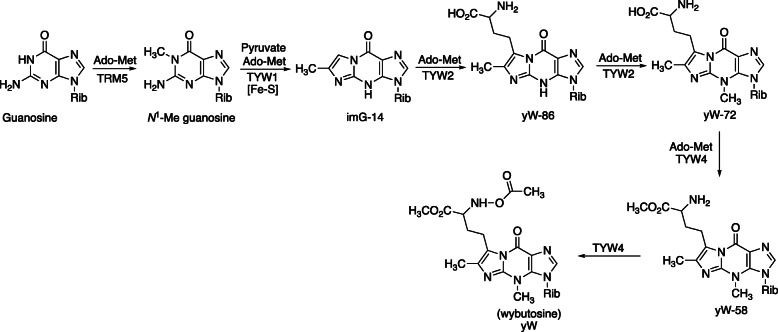
Biosynthetic pathway from guanosine (in rRNA) to wybutosine [13]. Ado-Met: *S*-adenosylmethionine

**Fig. 5:**
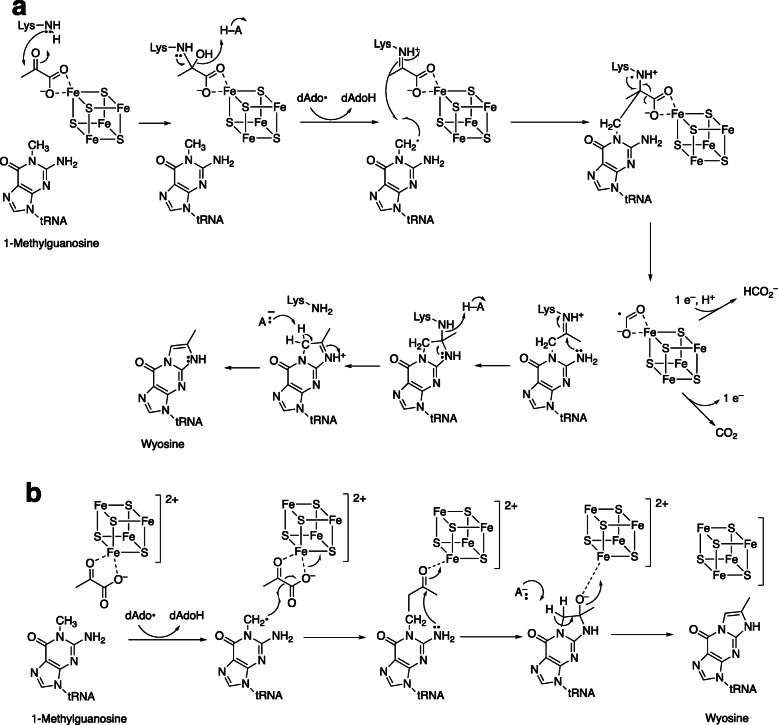
Proposed mechanisms for imidazoline ring formation in wyosine [14–16]

The biosynthesis of these etheno derivatives is complex and, in terms of metabolic strategies, expensive. A pathway for biosynthesis is shown in Fig. 4. The source of the extra two carbon atoms is pyruvate [14], and the catalysts involved are flavoproteins and radical *S*-adenosylmethionine (SAM) enzymes. The residual amino acid side chain (from methionine) is esterified and acetylated to form wybutosine (Fig. 4). Detailed proposals for formation of the imidazoline ring are presented in Fig. 5 [14–16].

## Etheno derivatives of cofactors

**Fig. 6:**
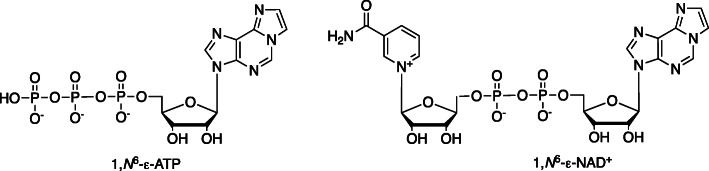
1,*N*^6^-ε-Ado derivatives of ATP and NAD^+^ [19–21]

In 1971 Kochetov et al. [17] reported that the reaction of 2-chloroacetaldehyde with 9-methyladenine and 1-methylcytosine yielded the *N*-methyl derivatives of 1,*N*^6^-ε-Ado and 3,*N*^4^-ε-Cyd (Fig. 1). Leonard and his associates used 2-chloroacetaldehyde to prepare 1,*N*^6^-ε-Ado and *N*^3^,4-ε-Cyd under mildly acidic conditions [18]. The latter group then used this approach to prepare the etheno derivatives of 3′-AMP, 5′-AMP, 3′,5′-cyclic AMP, ADP, and NAD^+^ [19]. These derivatives were active as cofactors in a number of enzyme systems and allowed for the analysis of binding parameters and other properties (Fig. 6) [20–22].

Unpaired bases in rRNA could also be modified with 2-chloroacetaldehyde, with up to 16 of the 23 adenines reacting and retention of 80% of the biological activity [23].

## Modification of DNA by products of vinyl chloride and other olefins generates etheno adducts

Vinyl chloride was found to produce unusual liver tumors in workers who handled this vinyl monomer in the polymer industry [24], and this cancer could be reproduced in rats [25]. Malaveille et al. [26] showed that the bacterial mutagenicity of vinyl chloride was dependent upon the addition of a liver microsomal system (containing cytochrome P450 (P450)) from rats, mice, or humans. Two of the suspected oxidation products of vinyl chloride, 2-chloroethylene oxide (vinyl chloride epoxide) and its rearrangement product 2-chloroacetaldehyde, were directly mutagenic [26], and both of these compounds reacted with free Ado to form 1,*N*^6^-ε-Ado [27].

Laib and Bolt [28] reported that 1,*N*^6^-ε-Ado was formed in the incubation of vinyl chloride with rat liver microsomes, poly-Ado, and NADPH, and 1,*N*^6^-ε-dAdo and 3,*N*^4^-ε-dCyd were formed in vitro under similar conditions [29]. 1,*N*^6^-ε-dAdo, 1,*N*^6^-ε-Ado, 3,*N*^4^-ε-dCyd, and 3,*N*^4^-ε-Cyd were identified as DNA and RNA adducts in livers of rats treated with ^14^C-vinyl chloride [29, 30].

Sattsangi et al. [31] had described the reaction of 2-chloracetaldehyde with guanosine in the synthesis of 1,*N*^2^-ε-Guo. The synthesis of *N*^2^,3-ε-Guo required blocking the O6 atom. Kúsmierek and Singer [32] also reported that 1,*N*^2^-ε-dGuo was formed in polynucleotides and DNA treated with 2-chloroacetaldehyde.

**Fig. 7:**
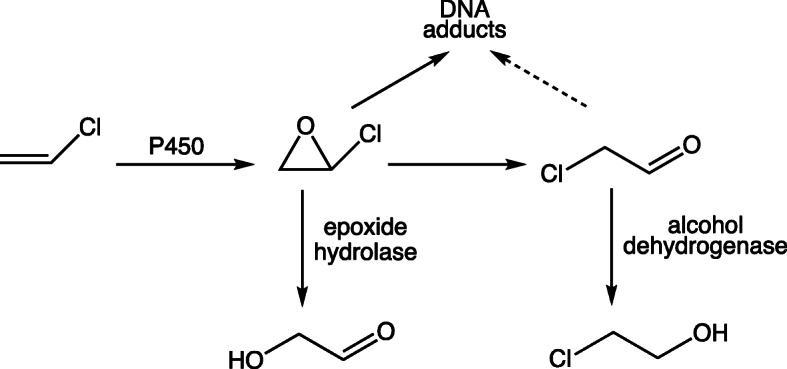
Oxidation of vinyl chloride to 2-chlorooxirane (vinyl chloride epoxide), rearrangement to 2-chloroacetaldehyde, and reaction with DNA (and RNA). The enzymes epoxide hydrolase and alcohol dehydrogenase (plus NADH) were used to attenuate each of the products and ascertain which is the major one involved in binding [33, 34]

Both 2-choroethylene oxide and its rearrangement product 2-chloroacetaldehyde are capable of reacting with nucleic acids to generate etheno adducts (Fig. 7, Table 1) [27]. Gwinner et al. [36] reported that 2,2′-dichlorodiethyl ether, which is hydroxylated and decomposes to 2-chloroacetaldehyde, did not yield etheno DNA adducts or *N*^7^-(2-oxoethyl) dGuo when given to rats nor did it produce the preneoplastic foci, a hallmark of vinyl chloride carcinogenesis. The results were congruent with in vitro results on the labeling of DNA by ^14^C-vinyl halides in microsomal reactions, with the epoxides and 2-haloacetaldehydes being quenched by the addition of enzymes (epoxide hydrolase and alcohol dehydrogenase) (Fig. 7) [33].

**Table 1:** DNA Adducts Formed in Reaction with 2-Chlorooxirane (Vinyl Chloride Epoxide)

Adduct^*a*^	Yield with 10 mM 2-chlorooxirane, pmol (μmol of DNA base)^− 1^	Method	Ref
*N*^7^-(2-oxoethyl)Gua	10,000	HPLC/fluorescence	[34]
1,*N*^6^-ε-dAdo	500	HPLC/fluorescence	[34]
HO-ethanoGua	24	HPLC/MS/MS	[35]
HO-ethanodGuo	29	HPLC/MS/MS	[35]
*N*^2^,3-ε-Gua	16	HPLC/fluorescence	[1]
3,*N*^4^-ε-dCyd	7	HPLC/fluorescence	[35]
1,*N*^2^-ε-Gua	~1^*b*^	HPLC/fluorescence	[1]
	2.5	HPLC/MS/MS	[35]

^a^The name of the adduct indicates whether the base or deoxyribonucleoside was assayed

^b^In other work, a 15 mg mL^− 1^ concentration of herring sperm DNA was used instead of 5 mg of calf thymus DNA mL^− 1^ in references [34], and a value of 6.5 pmol (μmol of DNA base)^− 1^ was obtained [1]

**Fig. 8:**
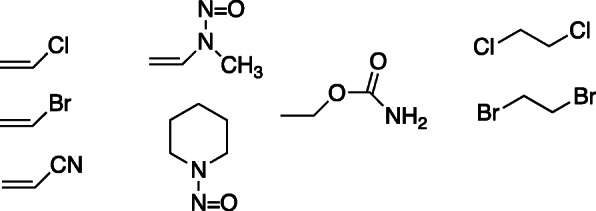
Chemicals known to lead to the formation of etheno adducts. The vinyl monomers undergo epoxidation to generate *bis*-electrophiles [37]. *N*-Nitrosopiperidine is α-hydroxylated to yield a product that breaks down to 4-oxo-2-pentenal and reacts to form propanone-1,*N*^2^-ε(d) Guo [3] (Fig. 2). The two ethylene dihalides can be hydroxylated to the *gem*-halohydrins, which then release HCl or HBr to yield 2-haloacetaldehydes [38]

Other chemical carcinogens were found to generate etheno DNA and RNA adducts, as a result of generation of *bis*-electrophiles (Fig. 8). Labeled 1,*N*^6^-ε-Ado and 3,*N*^4^-ε-Cyd were found in RNA of mice treated with ^14^C-ethyl carbamate [39], which is now understood to be oxidized to vinyl carbamate and then to the epoxide [39–41].

Acrylonitrile can be oxidized (by P450 2E1) to 2-cyanoethylene oxide, which reacts with Ado to form 1,*N*^6^-ε-Ado [42].

At least two nitrosamines can form etheno adducts. One is the epoxide derived from methyl, vinyl nitrosamine, which reacted with Ado to form 1,*N*^6^-ε-Ado [37]. A nucleophilic attack (adenosine) on the epoxide also generates methyl diazohydroxide, a methylating agent (which yielded *N*^7^-methyl dGuo). Hecht et al. [3] reported that the reaction of α-hydroxy-*N*-nitroso piperidine with DNA generated a 7-(2-oxopropyl) derivative of 1,*N*^2^-ε-dGuo. This product, proposed to be formed from 4-oxo-2-pentenal, has relevance to subsequent work on lipid peroxidation.

The characterization of human P450 2E1 allowed a number of cancer suspects to be examined as substrates. The enzyme was found to catalyze the oxidation of vinyl chloride, vinyl bromide, acrylonitrile, and vinyl carbamate to form 1,*N*^6^-ε-Ado (with Ado as a trap), presumably via the epoxides [43]. Ethylene dichloride and ethylene dibromide also yielded 1,*N*^6^-ε-Ado, presumably via oxidation to the 2-haloacetaldehydes. Subsequent studies in this [43] and several other laboratories have identified P450 2E1 as the major catalyst involved in the oxidation of small chemical carcinogens, especially vinyl monomers.

## Endogenous etheno DNA adducts

In the course of developing sensitive assays for DNA adducts, the Swenberg laboratory reported that *N*^2^,3-ε-dGuo was present in the DNA of livers of untreated rats [44]. This result was surprising in light of the growing number of vinyl monomers and related compounds that had been shown to form etheno adducts. The Bartsch and Chung laboratories showed that 1,*N*^6^-ε-dAdo and 3,*N*^4^-ε-dCyd could be generated during unsaturated lipid peroxidation [45–47]. The endogenous levels of some of the etheno DNA adducts are on the order of magnitude of other modified DNA bases associated with oxidative damage [44]. Further, levels of damage have been shown to be greatly increased (2- to 45-fold) by high fat diets and diseases of chronic inflammation [48].

**Fig. 9:**
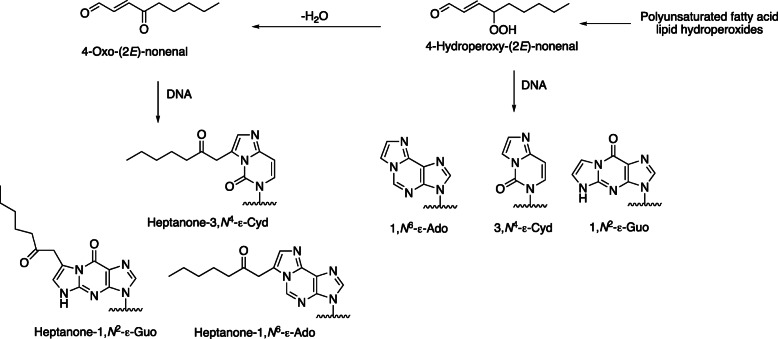
Generation of etheno adducts from peroxidation of unsaturated fatty acids [4]

**Fig. 10:**
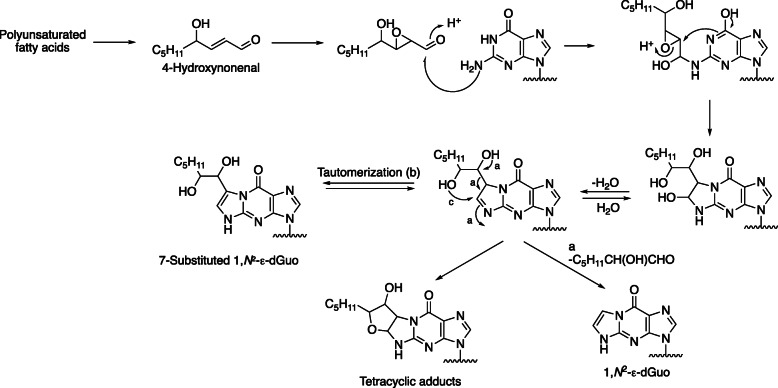
Reaction of dGuo with 4-hydroxynonenal epoxide leading to 1,*N*^2^-ε-dGuo and branched derivatives [49, 50]

Mechanisms of formation of etheno adducts are complex and can involve hydroperoxides, keto enols, and epoxides (Figs. 9 and 10). The proposed mechanism (Fig. 10) begins with carbinolamine/Schiff base formation with exocyclic amines, followed by reaction of a ring amine with the “second” electrophile (Fig. 10) [4, 49–54]. Some of the “branched” etheno DNA adducts, a 3-substituted 2-hexanone derivative of 3,*N*^4^-ε-dCyd [55] and a 1,*N*^2^-ε-dGuo derivative formed by *trans*-4-hydroxynonenal [56] have been shown to produce mutations in cells, as well as misinsertions in reactions catalyzed by individual DNA polymerases [57, 58]. Some of the etheno adducts derived from lipid peroxidation have been shown to lead to DNA interstrand crosslinks [59, 60].

A summary of analyses of etheno DNA adducts in untreated rats and humans is presented in Table 2. As can be noted, there is considerable variation, due in part to different methods of analysis. Most of the levels are a few adducts per 10^8^ nucleotides. It should be noted that 1,*N*^2^-ε-dGuo has not been measured in tissue samples. It was not detected in the rat liver work of Morinello et al. [64], even in vinyl chloride-exposed rats, and was considered unrelated to the tumors. However, the base (1,*N*^2^-ε-Gua) is excised by base excision repair and can be detected in human urine [71]. Levels of excretion were ~ 2-fold higher in smokers than non-smokers.

**Table 2:** Levels of Measured Endogenous Etheno DNA Adducts (see also [61])

DNA Source		Adducts/10^8^ nucleotides				Method	Reference
		1,*N*^6^-ε-dAdo	3,*N*^4^-ε-dCyd	1,*N*^2^-ε-dGuo	*N*^2^,3-ε-dGuo		
Rat	Liver	0.10–0.12	0.10–0.12			Immunoaffinity/^32^P labeling	[47]
	Liver				18–23	GC/MS	[47]
	Liver	120	560			HPLC/^32^P labeling	[62]
	Liver	0.011–0.012	0.016–0.017			Immunoaffinity/^32^P labeling	[63]
	Lung	0.4–0.5	1.8–3.8			Immunoaffinity/^32^P labeling	[63]
	Kidney	0.7–0.8	2.1–2.8			Immunoaffinity/^32^P labeling	[63]
	Lymphocytes	1.1	1.2			Immunoaffinity/^32^P labeling	[63]
	Hepatocytes				1.4	Immunoaffinity/GC/MS	[64]
	Hepatocytes			ND^*a*^		Immunoaffinity/GC/MS	[65]
Human	Hepatocytes				2.0	LC/MS	[66]
	Oral cells	0.77	0.61			LC/MS	[67]
	Placenta	74–84				LC/MS	[68]
	Placenta	1.1				LC/MS	[69]
	Pancreas	1.8	1.2			Immunoaffinity/^32^P labeling	[70]

^a^*ND* Not detected (< 4)

## Chemical mechanisms of formation of etheno adducts

Reactions of *bis*-electrophiles can be complex in that there are two sites of reaction with nucleophiles (e.g., DNA). Moreover, there may be a series of possible electrophiles due to instability of some, e.g. epoxides.

As already mentioned (Fig. 7), early studies with vinyl chloride and vinyl bromide showed that epoxide hydrolase was more effective in attenuating the binding of radioactivity from either vinyl halide to DNA in microsomal incubations [33], implicating 2-haloethylene oxides as the reactive species, instead of 2-haloacetaldehydes. These results were opposite of those experiments in which protein binding was measured [72] and can be rationalized in the context of hard (DNA) and soft (thiol) nucleophiles reacting with different electrophiles.

The reaction of *N*^6^-methylAdo with 2-chlorooxirane was two orders of magnitude faster than with 2-chloroacetaldehyde [38]. The yields of 1,*N*^6^-ε-dAdo and *N*^2^,3-ε-dGuo were also two orders of magnitude higher with 2-chlorooxirane than 2-chloroacetaldehyde, and (as in Fig. 7) epoxide hydrolase was more effective than alcohol dehydrogenase in attenuating the levels of both adducts formed in DNA in incubations with vinyl chloride and rat liver microsomes [1, 34].

**Fig. 11:**
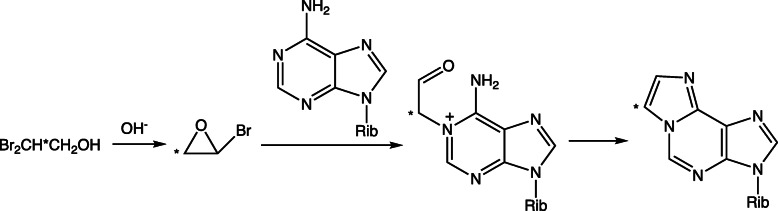
Mechanism of reaction of a 2-halooxirane with Ado to form 1,*N*^6^-ε-Ado [38]. * indicates a ^13^C label

**Fig. 12:**
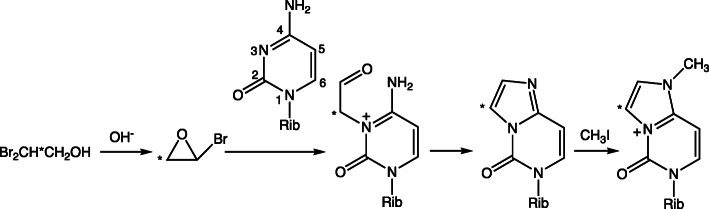
Mechanism of reaction of a 2-halooxirane with Cyt to form 3,*N*^4^-ε-Cyt [38]. * indicates a ^13^C label

**Fig. 13:**
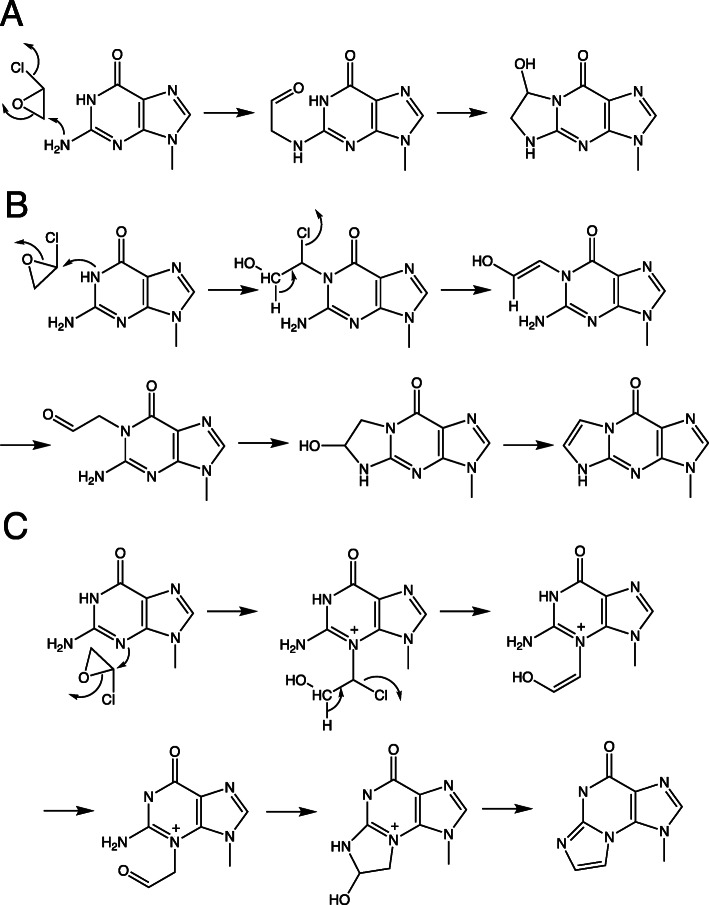
Mechanism of reaction of 2-chlorooxirane with Guo to form **a** 7-OH-1,*N*^2^-ε-Guo, **b** 1,*N*^2^-ε-Guo, and **c** *N*^2^,3-ε-Guo [1]

Mechanisms for the reaction of 2-haloethylene oxides with Ado (Fig. 11) and Cyd (Fig. 12) have been elucidated using ^13^C NMR spectroscopy [38]. The approach involved the slow generation of 2-bromoethylene oxide in situ from 2,2-dibromoethanol at pH 9.2. The ^13^C labeling patterns with the 1,*N*^6^-ε-Ado and 3,*N*^4^-ε-Cyd showed that the initial reaction is of a ring nitrogen (N1 of Ado or N3 of Cyd) with the unsubstituted methylene carbon of the 2-haloethylene oxide [38]. Similar studies were done with Guo (Fig. 13) [1]. As with Ado and Cyd, the formation of 1,*N*^2^-ε-Guo is explained by the reaction of the (ring) N1 nitrogen on the methylene carbon of the 2-haloethylene oxide. However, the formation of *N*^2^,3-ε-dGuo is more complex, and we concluded that the N3 (ring) nitrogen of dGuo reacts first with the halogen-substituted carbon of the 2-haloethylene oxide, with subsequent reaction of the (formed) aldehyde with the exocyclic (N2) nitrogen, followed by dehydration of the carbinolamine [1].

**Fig. 14:**
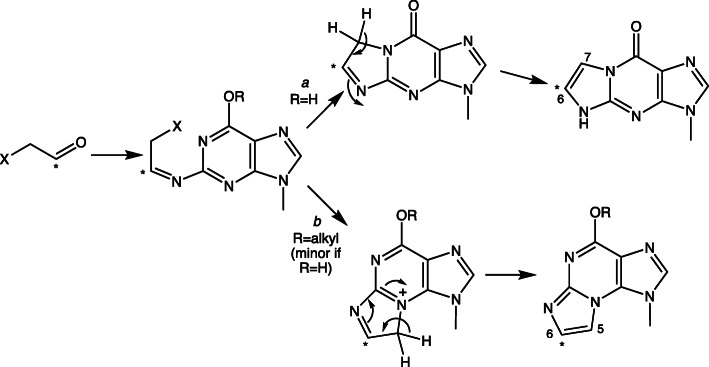
Mechanism of reaction of 2-haloacetaldehydes with Guo to form **a** 1,*N*^2^-ε*-*Guo and **b** *N*^2^,3-ε-Guo [73]

When 2-bromoacetaldehyde reacted with Guo, the ^13^C labeling pattern indicated that the initial reaction was that of the aldehyde to form a Schiff base, followed by attachment of the N1 atom to form 1,*N*^2^-ε-Guo or the N3 atom to form *N*^2^,3-ε-Guo (Fig. 14) [73]. Kúsmierik and Singer [74] had reported that the reaction of 2-chloroacetaldehyde with Ado or Cyd yielded quasi-stable carbinolamine intermediates (“hydrates”). The reaction of dGuo with glycidaldehyde to form 1,*N*^2^-ε-dGuo also proceeds via initial Schiff base reaction of the 1-amino group with the aldehyde [75].

We conclude that 2-haloethylene oxides are much more reactive than 2-haloacetaldehydes in reacting with DNA and RNA (Fig. 7). The results have biological relevance in that other chemicals that can generate 2-haloacetaldehydes after metabolism are not very carcinogenic [36] (Fig. 7). Treatment of DNA with 2-chlorooxirane yields a series of products, including 7-hydroxyethano-dGuo, a stable hemiaminal (Fig. 2). The products were formed in the order *N*^7^-(2-oxoethyl) dGuo > > 1,*N*^6^-ε-dAdo > 7-hydroxyethano dGuo > *N*^2^,3-ε-dGuo > 3,*N*^4^-ε-dCyd > 1,*N*^2^-ε-dGuo (Table 1) [35].

In the course of studies on the mechanisms of formation of 1,*N*^2^-ε-(d) Guo and *N*^2^,3-ε-(d) Guo [1], some anomalous behavior was noted when isotopic labeling studies were attempted with deuterium. The H-5 proton of *N*^2^,3-ε-Guo was lost upon heating under mildly acidic conditions and explained the inability to prepare [5-^2^H]-*N*^2^,3-ε-Guo from ClC^2^H_2_CHO (ClCD_2_CHO). The H-5 proton of *N*^2^,3-ε-Guo and the H-7 proton of 1,*N*^2^-ε-Guo were selectively exchanged at either pH 7.7 or 9.2. Mechanisms involving tautomeric exchange have been proposed [1]. Some of these results may explain sensitivity to acid and base in early studies on the tRNA Y-bases (vide supra, Fig. 3) [7, 9].

## Effect of etheno adducts on oligonucleotide structures

Some of the early work involved treatment of tRNA with 2-chloroacetaldehyde [23] but the results were rather non-descript. An interesting finding was the reaction of chloroacetaldehyde with Z-(left-handed) DNA [76]; adenines (*syn* conformation) preferentially reacted compared to cytosines (although etheno adducts were not characterized). In the reaction of 2-chloroacetaldehyde with model polynucleotides, hydrogen bonding in double-stranded structures was important in the formation of 1,*N*^2^-ε-Gua [32]. The formation of *N*^2^,3-ε-Gua was relatively independent of whether the DNA was single- or double-stranded.

In 15-mer oligomeric duplexes, the pairing of 3,*N*^4^-ε-dCyd opposite dGuo, not surprisingly, destabilized the helix but not as much as a T:G mispair [77].

Apparently only two X-ray crystal structures have been published with etheno adducts present, and both are self-complementary. One has 1,*N*^6^-ε-dAdo:dGuo pairing [78] and the other has 3,*N*^4^-ε-dCyd:dGuo pairing [79]. As expected, both have lost the normal base pairing.

More NMR studies have been published on pairing of etheno bases in oligonucleotides [80–89]. The majority of the NMR studies with 3,*N*^4^-ε-dCyd have it paired with dGuo, with normal H-bonding blocked and a hydrogen bond involving the O2 atom of 3,*N*^4^-ε-dCyd and the N1 atom of dGuo, as in an X-ray structure, and similar to a T:G wobble pair. Both nucleotides in the 3,*N*^4^-ε-dCyd:dGuo pair were in the *anti* configuration but in a 3,*N*^4^-ε-dCyd:dThd pair the 3,*N*^4^-ε-dCyd lesion was *syn* and dThd was *anti* [80]. The alignment of 1,*N*^6^-ε-dAdo with dThd was nonplanar [85]. In a 1,*N*^6^-ε-Ado:dGuo pair the 1,*N*^6^-ε-Ado was *syn* but dGuo was *anti* [86], as in the crystal structure [78].

Several NMR studies of 1,*N*^2^-ε-dGuo oligonucleotides have been published, at varying pH values [87–89]. The results are indicative of Hoogsteen pairing and a blend of conformations at neutral pH. A structure with 1,*N*^2^-ε-dGuo opposite a 1-base deletion showed increased duplex stability and can be considered as supportive of the tendency of 1,*N*^2^-ε-dGuo to cause − 1 frameshifts [90].

1,*N*^6^-ε-Ado and 3,*N*^4^-ε-Cyd have been positioned in ribooligonucleotides and shown to cause destabilization of complexes with RNA and DNA complements [91].

## Interactions of etheno DNA adducts with DNA polymerases: structural and functional studies

One of the most interesting aspects of studying DNA adducts is understanding the details of how an individual lesion causes miscoding. This has been a long-term goal but is not an easy one to accomplish, for a number of reasons.

The synthesis of oligonucleotides containing an adduct at a specific site can be problematic. The general approach is to prepare the modified base as a nucleoside and insert it using chemical synthesis. The adduct must be stable to the conditions of protection and deprotection. The oligonucleotide containing the adduct must be stable and also be very pure, especially if introduced into a cellular system, where the progeny of impurities are probably not discernible.

The question arises as to what to look at for miscoding. There is attraction to the use of a cellular system, as first developed by Essigmann and his associates in 1984 [92]. However, there are a number of aspects to consider. Should one use bacterial or mammalian cells? The presence of DNA repair systems can be problematic, in terms of attenuating responses (but cells with repair deficient backgrounds may be useful). There is also the issue, generally ignored, that almost all of the studies in this area have been “extra-chromosomal,” i.e. the vectors (plasmids) may not be copied in the same manner as endogenous DNA adducts due to the use of different polymerases and accessory factors (for an exception see our work with 1,*N*^2^-ε-dGuo in [93]). *Escherichia coli* has five DNA polymerases and humans have at least 19 with some kind of polymerization activity, so how does one discern which is involved? One way is to compare in vitro assay results with different (purified) polymerases, i.e. specificity constants (*k*_cat_/*K*_m_). At the cellular level, it is now relatively easy to use CRISPR systems to make mammalian cell lines deficient in each polymerase and then compare mutation frequencies.

In order to define details of miscoding at a biochemical level, it is necessary to use individual DNA polymerases. In the early work, the use of viral, bacterial, or archaebacterial polymerases was popular but might not have been reflective of eukaryotic systems. Today most of the attention has been given to the so-called translesion synthesis (TLS) DNA polymerases (pols), especially the human ones (in the Y-Family, i.e., η, ι, κ, Rev1). There are two major approaches to understanding the actions of DNA polymerases, functional and structural.

Functional assays are largely focused on enzyme kinetics. Most of the work is with insertion into primers bound to templates containing an adduct, but some studies are done with dNTP extension past template-DNA adduct:primer mispairs. If the DNA adduct is very blocking, then steady-state kinetics analysis may be appropriate. However, in some cases it is preferable to use pre-steady-state kinetic analysis, especially if burst kinetics are observed [94].

**Fig. 15:**
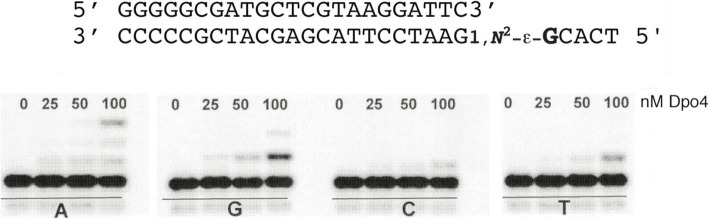
Incorporation of dNTPs across from 1,*N*^2^-ε-dGuo in a primer and extension beyond. The indicated primer template complex was incubated with the DNA polymerase Dpo4 and each dNTP (A, G, C, T). The primer contained a 5′-^32^P label, and the products of each reaction was analyzed by denaturing gel electrophoresis [90]

**Fig. 16:**
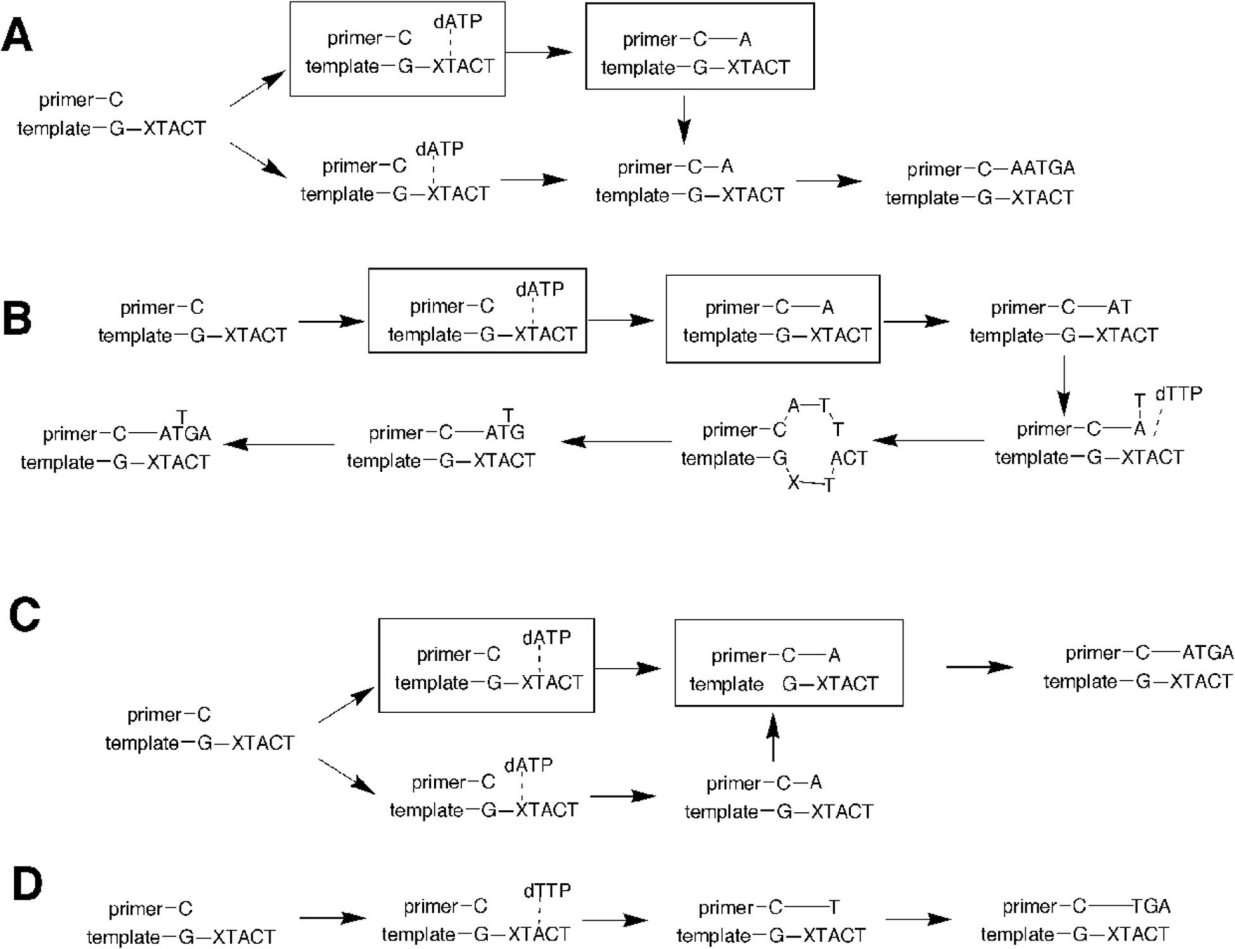
Assignment of pathways for insertion and extension for a mixture of dNTPs and Dpo4, as analyzed by LC-MS [90]. The percentages indicate the fraction of each product formed. X-ray crystal structures of the complexes indicated in boxes were obtained

Defining what the products are may not be trivial, as emphasized in our study with *Sulfolobus solfataricu*s Dpo4 and 1,*N*^2^-ε-dGuo (Fig. 15) [90] (vide infra). There are several approaches to defining the products of polymerase extension of primer:template complexes, which can be complex [95]. One is LC-MS sequence analysis (Fig. 16), which is relatively straightforward and still remains the method of choice in our own laboratory [90, 96, 97]. Others are the “REAP” and “CRAB” methods developed by Essigmann’s laboratory [98]. The last, which has become feasible in recent years, is total sequencing [99].

The other aspect, structure, involves X-ray crystallography, which has proven to be very feasible for TLS DNA polymerases and DNA adducts. The only realistic bacterial DNA polymerases for crystallography have been pol I, II, and IV [100–102]. Pol III and pol V, although they can have roles with DNA adducts, are too complex. The Y-Family TLS polymerase *S. solfataricus* Dpo4 has been popular as a model. Many structures of the core elements of human pol η, ι, and κ with adducts have now been reported, and in many cases the details have been quite revealing about mechanisms. Structural studies with the replicative DNA pols δ and ε (and *E. coli* pol III and V) are not very realistic due to the number of subunits. Although pol β has been a popular model for studies, this is really a gap-filling DNA polymerase and its relevance in studies on primer extension (or even insertion with a gap opposite a DNA adduct) is questionable.

The final point to make is that different polymerases may vary in terms of how they deal with a single DNA adduct. This is exemplified in work with *O*^6^-methyl dGuo [103, 104] and in the case of 1,*N*^2^-ε-dGuo (Table 3).

**Table 3:** *N*^2^,3-ε-dGuo vs. 1, *N*^2^-ε-dGuo [90, 105–108]

		
Polymerase	Template	dNTP	***k***_**cat**_/***K***_**m**_ (μM^− 1^ min^− 1^)	***f***	Template	dNTP	***k***_**cat**_/***K***_**m**_ (μM^− 1^ min^− 1^)	***f***
*E. coli* pol I KF (exo¯)	3′-G*TA-	*N*^2^,3-ε*-*dG:C	0.23	1		1,*N*^2^-ε-dG:C	0.0081	1
		*N*^2^,3-ε-dG:T	0.24	1	3′-G*TG-	1,*N*^2^-ε-dG:G	0.0087	1.1
						1,*N*^2^-ε-dG:A	0.0016	0.2
*S. solfotaricus* Dpo4	3′-G*TA-	*N*^2^,3-ε-dG:C	0.025	1	3′-G*TA-	1,*N*^2^-ε-dG:C	0.00006	1
		*N*^2^,3-ε-dG:T	0.0054	0.22		1,*N*^2^-ε-dG:A	0.0008	14
Human pol κ	3′-G*TA-	*N*^2^,3-ε-dG:C	0.022	1	3′-G*TA-	1,*N*^2^-ε-dG:C	0.0012	1
		*N*^2^,3-ε-dG:T	0.0081	0.37		1,*N*^2^-ε-dG:T	0.0012	1
Human pol ι	3′-G*TA-	*N*^2^,3-ε-dG:C	0.0017	1	3′-G*TA-	1,*N*^2^-ε-dG:C	0.0017	1
		*N*^2^,3-ε-dG:T	0.0012	0.71		1,*N*^2^-ε-dG:T	0.016	9.6

G*: *N*^2^,3-ε-dG or 1,*N*^2^-ε-dG; *f* = (*k*_cat_/*K*_m_)_incorrect_/(*k*_cat_/*K*_m_)_correct_ 
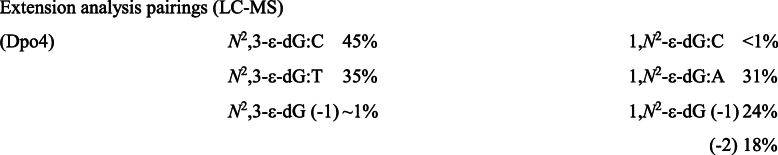

Relatively few studies have been done with 3,*N*^4^-ε-dCyd. *E. coli* pol I (Klenow fragment) inserted dAMP and dTMP opposite 3,*N*^4^-ε-dCyd [109]. The same misinsertions were seen with mammalian pol α, β, and δ [110] and in cellular *E. coli* and monkey kidney cells [111]. To our knowledge, no polymerase crystal structures with 3,*N*^4^-ε-dCyd have been reported, only those with a modified oligonucleotide in the absence of polymerase [79].

**Fig. 17:**
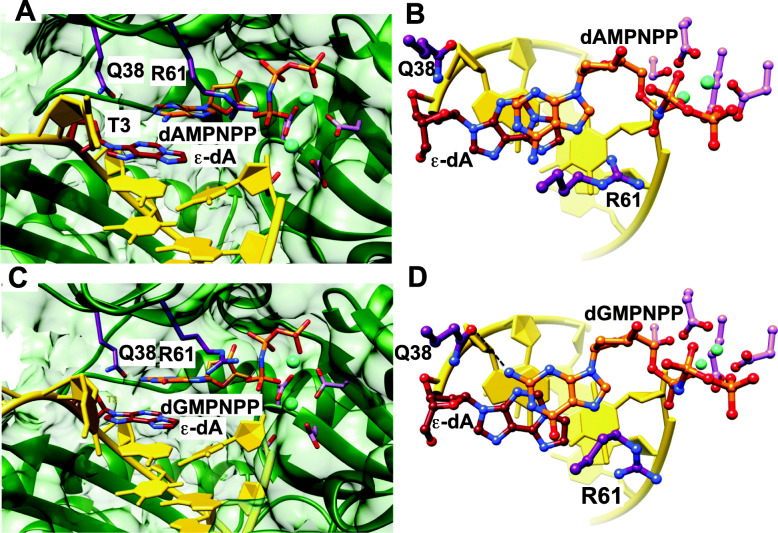
Staggered arrangements of incoming purine nucleoside triphosphates and 1,*N*^6^-ε-dAdo in two pol η insertion stage complexes [118]. **a** active site conformation in the complex with dAMPNPP opposite 1,*N*^6^-ε-dAdo, viewed into the DNA major groove; **b** rotated by 90° and viewed perpendicular to the adenine plane. **c** Active site conformation in the complex with dGMPNPP opposite 1,*N*^6^-ε-dAdo, viewed into the DNA major groove, and **d** rotated by 90° and viewed perpendicular to the guanine plane. Selected active site residues are colored by atom with carbon atoms shown in maroon (1,*N*^6^-ε-dAdo), orange (incoming nucleotide), purple (Arg-61 and Gln-38 from the finger domain), or magenta (Asp/Glu coordinating to Mg^2+^; cyan spheres)

With regard to 1,*N*^6^-ε-dAdo, Singer’s laboratory reported that all four of the dNTPs could be incorporated opposite this lesion by *E. coli* pol I and that the results were influenced by the choice of polymerase and the sequence [112, 113]. The bases Ade, Cyt, and Gua were all reported to be misinserted in various extrachromosomal cellular misincorporation systems [114–117]. Levine et al. [117] had reported that human pol η was 100-fold more active than pol κ in replication past 1,*N*^6^-ε-dAdo, and our laboratory found that replication past 1,*N*^6^-ε-dAdo was dominated by incorporation of purines (dAdo, dGuo) and by extensive − 1 frameshifts (Fig. 17) [118]. Frameshifts are not generally observed in simple primer-extension studies but are readily detected by LC-MS analysis [90, 118]. X-ray crystal structures indicated that the incoming dATP and dGTP were not paired with 1,*N*^6^-ε*-*dAdo but were in a staggered configuration relative to 1,*N*^6^-ε-dAdo, opposite a 5-dThd in the sequence and explaining the proclivity for frameshifts [118]. When a dTTP analog was positioned opposite 1,*N*^6^-ε-dAdo, the adduct was in the *syn* configuration. In a separate study, the Agarwal group [119] showed that pol ι used Hoogsteen base pairing to promote synthesis beyond 1,*N*^6^-ε-dAdo.

The 1,*N*^2^-ε-dGuo adduct completely blocks the normal Watson-Crick pairing face (Fig. 1) and has been studied extensively. Early studies with *E. coli* pol I and II, HIV reverse transcriptase, and rat pol β showed normal incorporation and the insertion of dATP and dGTP [120]. In *E. coli*, all three mispaired bases were inserted (T, A, G) [121]. Stable integration of an oligonucleotide in the chromosome of Chinese hamster ovary cells led to a number of base pair mutations (due mainly insertions of A and T opposite 1,*N*^2^-ε-Guo), plus substitutions removed from the site of DNA damage and some unexplained rearrangements [93].

**Fig. 18:**
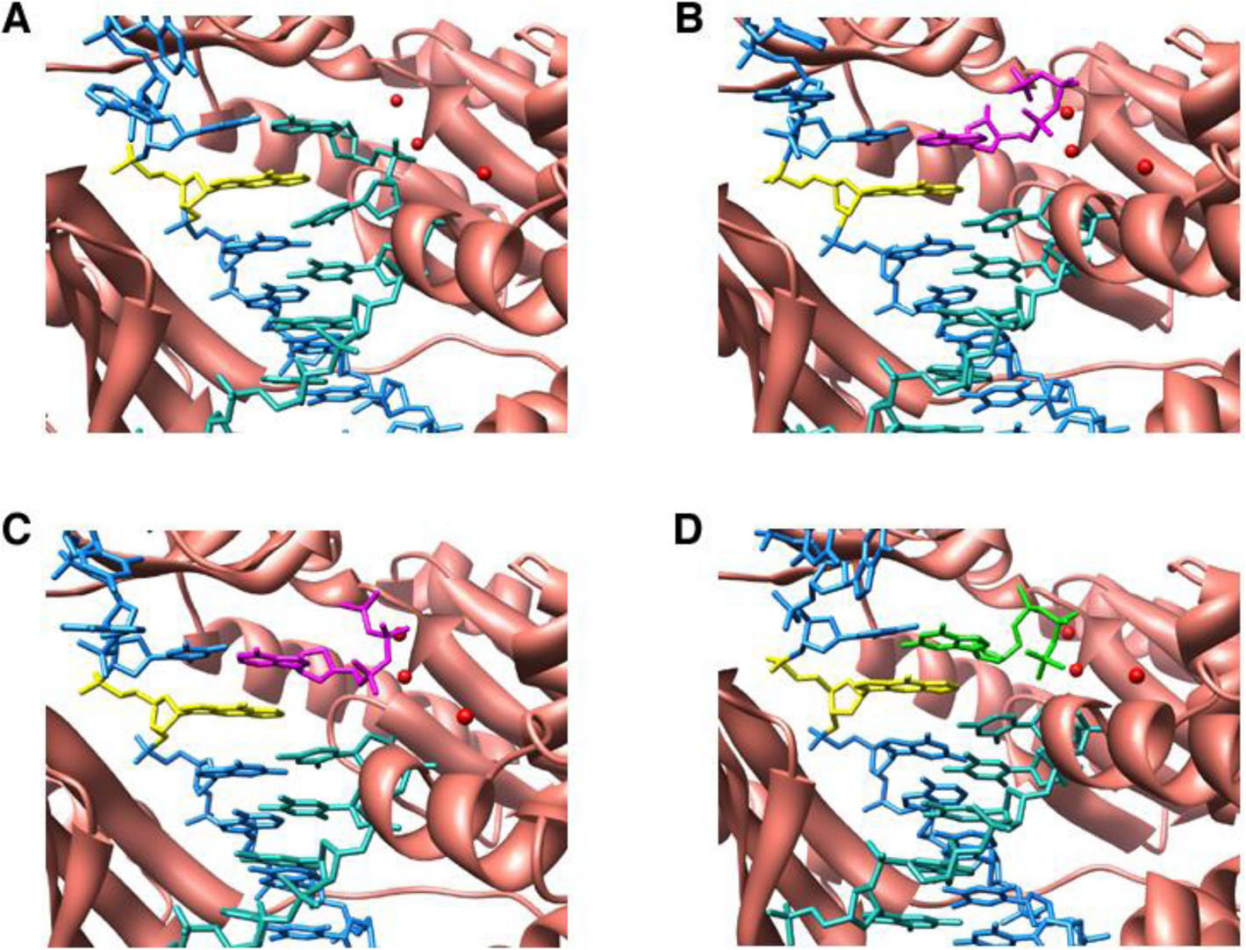
Close views of active site regions of Dpo4 crystal structures [90]. **a** Dpo4•DNA•Ca^2+^ (*X* = 1,*N*^2^-ε-dGuo and *Z* = T). **b** Dpo4•DNA•dATP•Ca^2+^ (*X* = 1,*N*^2^-ε-dGuo and *Z* = T). **c** Dpo4–3. Dpo4•DNA•ddATP•low Mg^2+^ (*X* = 1,*N*^2^-ε-dGuo and *Z* = T). **d** Dpo4–4. Dpo4•DNA•Ca^2+^ (*Z* = C). The color codes for protein and DNA are: dATP and ddATP are drawn in *pink*, ddGTP in *green*, and divalent metal ions are shown as *red spheres*

A study with 1,*N*^2^*-*ε*-*dGuo and *S. solfataricus* Dpo4 yielded some initially confusing results, in that reaction of a primer:(1,*N*^2^-ε-dGuo) template complex led to the incorporation of three dATPs (Fig. 15), which seemed highly unusual in light of the sequence context, even if dATP were incorporated opposite 1,*N*^2^-ε-dAdo. LC-MS approaches were developed to analyze the product, which proved to be a mixture of four major products. The content of each could be approximated by LC-MS (Fig. 16). A scheme could be drawn to explain the − 1 and − 2 frameshifts, plus the other products (Fig. 16). X-ray structures (Fig. 18) of all oligonucleotide pairing possibilities shown in boxes (Fig. 16) could be solved [90]. A major structure is a “Type II” complex in which the polymerase skips the adduct and pairs with the next base [90]. Extension of the work to human DNA polymerases [105] showed that pol δ was completely blocked by the presence of 1,*N*^2^-ε-dGuo and that pol η was the most active in copying past 1,*N*^2^-ε-dGuo. pol η preferred to insert dGTP > dATP > dCTP. Apparently the 1,*N*^2^-ε-dGuo:dGuo base pairs are extended but others not so well. More recently, X-ray crystal structures have been obtained with C and A placed opposite 1,*N*^2^-ε-dGuo in human pol η [106]. Mass spectral analysis of fully-extended products revealed the misinertion of G (85%) opposite 1,*N*^2^-ε-dGuo lesion. Importantly, the post-lesion extension from the correct nucleotide pair (1,*N*^2^-ε-dGuo:dCyd) was not observed, indicating that the “correct” pair was retarded regarding extension past the lesion by pol η [106].

Both 6- and 7-hydroxy derivatives of 1,*N*^2^-ethano-dGuo (Fig. 2) are relevant biologically (Fig. 10) and have been examined for miscoding with some individual DNA polymerases [2, 120] and, in the case of 7-hydroxy derivative in *E. coli* cells [121]. The results are similar to those obtained with 1,*N*^2^-ε-dGuo, with some differences.

The miscoding potentials of two substituted 1,*N*^2^-ε-dGuo adducts derived from lipid peroxidation have also been examined. Moriya’s group studied 3-(2-heptanone)-3,*N*^4^-ε-dCyd and showed incorporation of dTTP and dATP in mouse fibroblasts [55]. The insertion of dTTP could be catalyzed by pol η, κ, or ι but insertion of dATP was attributed to a different, unknown polymerase, which could not extend beyond the insertion. Extension beyond the mispair was attributed to a pol ξ-Rev1 complex [55]. In a study with *S. solfataricus* Dpo4, the Rizzo laboratory found miscoding behavior of 7-(2-oxoheptyl)-1,*N*^2^-ε-dGuo similar, but not identical, to 1,*N*^2^-ε-dGuo [57].

Although *N*^2^,3-ε-dGuo is more abundant in DNA than 1,*N*^2^-ε-dGuo (Table 1), it has been studied less. One of the major reasons is the technical difficulties in placing this lesion in an oligonucleotide because the glycosidic bond is unstable to hydrolysis [122]. Singer et al. [123, 124] were able to incorporate the nucleoside triphosphate into an oligonucleotide template and show misincorporation opposite the lesion with HIV-1 reverse transcriptase. Insertion of 1,*N*^2^-ε-dGTP opposite template T was also analyzed [124].

**Fig. 19:**
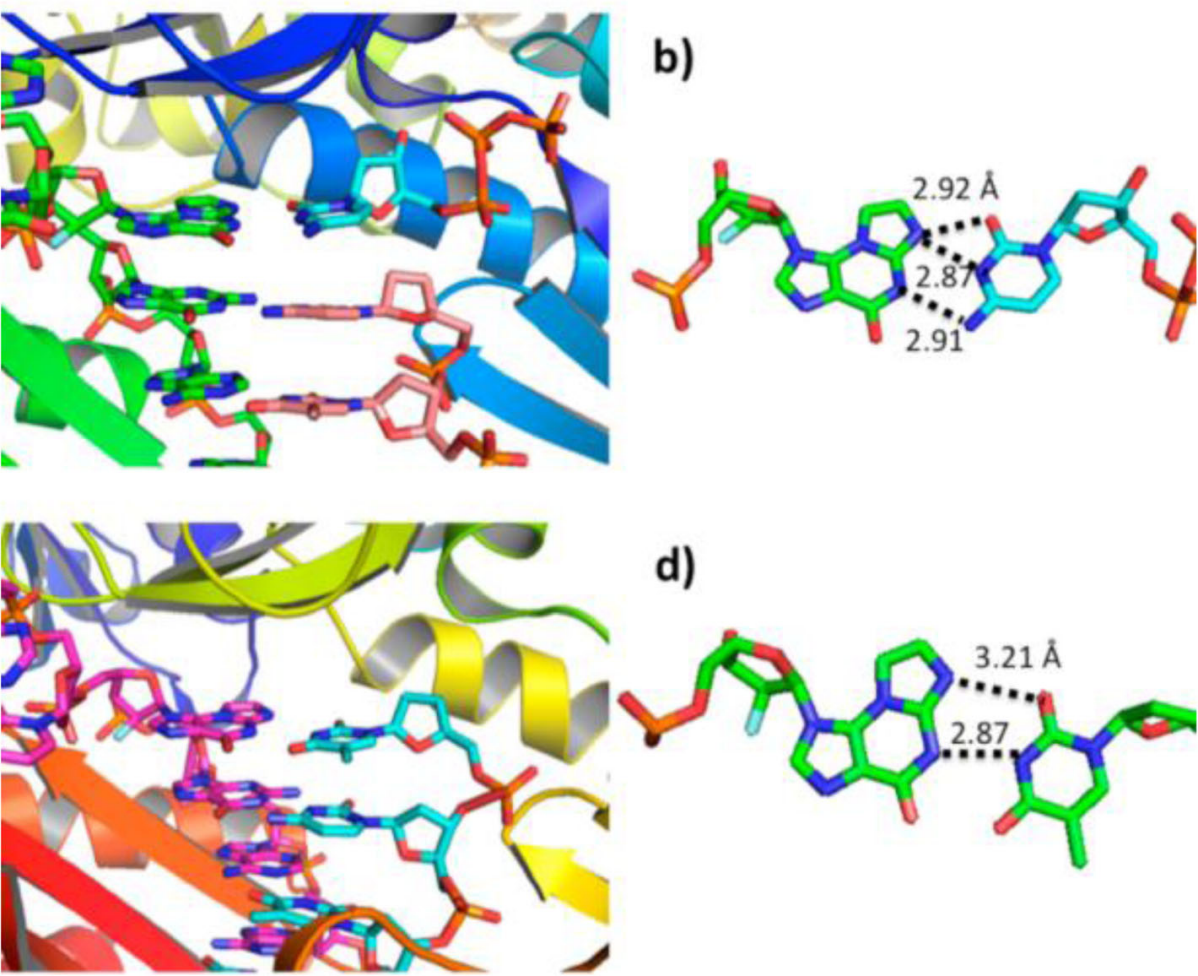
Crystal structures of Dpo4•*N*^2^,3-ε-dGuo-DNA complex (Z = C in the template) [107]. **a** Ternary complex of dCTP•*N*^2^,3-ε-dGuo and **b** the orientation of the bases with proposed hydrogen bonding mechanism. **c** Binary complex of ddT•*N*^2^,3-ε-dGuo and **d** the orientation of the bases with proposed hydrogen bonding mechanism

In order to circumvent the issue of the glycosidic instability of *N*^2^,3-ε-dGuo, we used an isostere approach previously applied to *N*^7^-methylguanine [125]. Deoxyribose was replaced with 2′-fluoroarabinose, i.e. the addition of fluorine at the 2′ carbon of the sugar ring adds electronegativity and destabilizes the transition state for glycosidic cleavage. The half-life of the adduct at 37 °C was increased to 23 days and allowed detailed structural and biochemical studies to be done with both *S. solfataricus* Dpo4 and other enzymes, including human pol ι [107, 108]. The overall differences in the behavior of 1,*N*^2^-ε-dGuo and *N*^2^,3-ε-dGuo are shown in Table 3. For *N*^2^,3-ε-dGuo the fidelity (with Dpo4) is considerably higher and there are few frameshifts. With Dpo4, the crystal structure of the 1,*N*^2^-ε-dGuo:dCTP pair is pseudo-Watson Crick and the 1,*N*^2^-ε-dGuo:dTTP pairing is “wobble-like” (Fig. 18) [107]. With pol ι, there was Hoogsteen-like pairing, with two hydrogen bonds in the *N*^2^,3-ε-dGuo:dCTP pair and only one in the *N*^2^,3-*ε*-dGuo:dTTP pair (Fig. 19) [108]. dTTP insertion was the major misincorporation event with all of the human Y-Family TLS polymerases examined, with pol ι having the highest frequency [108].

The dTTP pairing with *N*^2^,3-ε-dGuo is also consistent with a dominant G to A transition pattern seen with *N*^2^,3-ε-dGuo in *E. coli* [99]. Collectively, the work is relevant in that G to A transitions were the dominant mutations observed in vinyl chloride-associated liver tumors [126]. Perhaps the mystery of vinyl chloride and cancer etiology has finally been revealed.

## Repair of etheno DNA adducts

The repair of etheno adducts has long been studied, going back > 30 years. Swenberg et al. [127] reported that 1,*N*^6^-ε-dAdo, 3,*N*^4^-ε-dCyd, and *N*^2^,3-ε-dGuo were all persistent in rat liver. Moreover, the ratios of the adducts (Table 2) differ from what is observed upon reaction of 2-chloroethylene oxide with DNA (Table 1), suggesting different half-lives of individual adducts.

Oesch et al. [128] reported the release of 1,*N*^6^-ε-dAdo and *N*^2^,3-ε-dGuo from chloroacetaldehyde-treated DNA by an extract of rat brain cells. Rydberg et al. [129, 130] reported glycosylase activity towards 1,*N*^6^-ε-dAdo in human cell-free extracts, and Singer et al. [131] reported that human *N*^3^-methyl Ade-DNA glycosylase could act on 1,*N*^6^-ε-dAdo. Although Singer’s group reported that a single human DNA glycosylase could release 1,*N*^6^-ε-dAdo, 3,*N*^4^-ε-dCyd, 1,*N*^2^-ε-dGuo, and *N*^2^,3-ε-dGuo [132], they subsequently reported that 1,*N*^6^-ε-dAdo and 3,*N*^4^-ε-dCyd were excised by separate (human) glycosylases [133]. Later, Saparvaev et al. [134] reported that *E. coli* mismatch-specific uracil-DNA glycosylase and human alkylpurine-DNA-N-glycosylase can excise 1,*N*^2^-ε-dGuo. Ethano Cyt and Ade adducts (saturated) are also substrates for *E. coli* glycosylases [135].

**Fig. 20:**
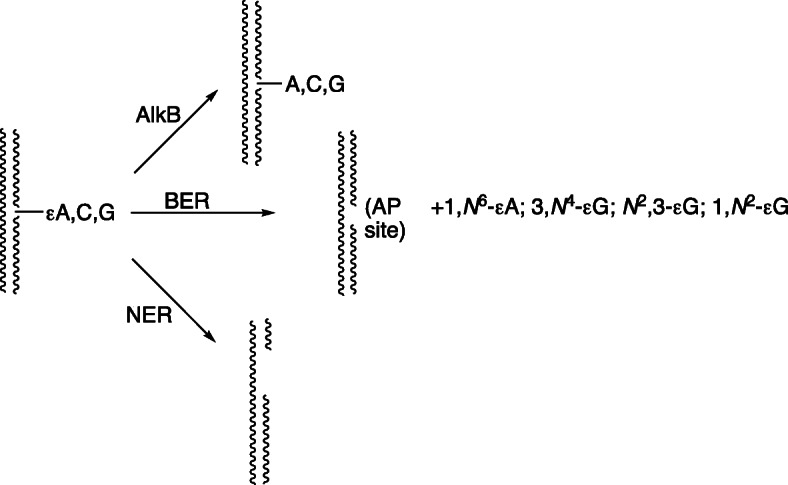
Mechanisms of repair of etheno DNA adducts. AlkB is a direct pathway catalyzed the bacterial dioxygenase AlkB and mammalian homologues. BER: base excision repair. NER: nucleotide excision repair

Repair of DNA etheno adducts is not restricted to glycosylases, in that they are also subject to direct reversal and nucleotide excision repair (NER) (Fig. 20). Evidence has also been reported that NER can be involved in DNA repair, at least for 1,*N*^6^-ε-dAdo and 3,*N*^4^-ε-dCyd (Fig. 20) [136, 137].

**Fig. 21:**
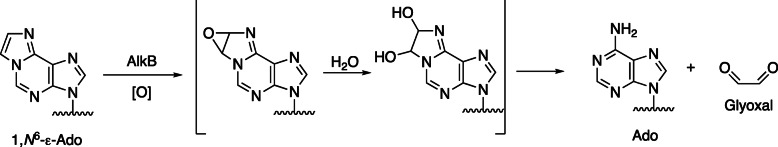
Direct repair of 1,*N*^6^-ε-dAdo by AlkB [138]. Putative intermediates are in brackets

AlkB is an α-ketoglutarate-dependent dioxygenase that was discovered in bacteria for its ability to catalyze hydroxylation and removal of alkyl groups at the ring nitrogens of DNA bases. The Essigmann laboratory showed that the enzyme, and its mammalian orthologues, can catalyze the direct removal of the two carbons of etheno bases (1,*N*^6^-ε-dAdo; 3,*N*^4^-ε-dCyd) as glyoxal (Fig. 21). Tudek and associates compared the removal of 1,*N*^6^-ε-dAdo, 3,*N*^4^-ε-dCyd, and 1,*N*^2^-ε-dGuo by nine bacterial AlkB and two human AlkB homologues [139]. Two bacterial AlkB-type enzymes had no activity. Three removed all three of the etheno adducts, and two of these did not act on any alkyl DNA adducts. Another three removed 1,*N*^6^-ε-dAdo and 3,*N*^4^-ε-Cyd but not 1,*N*^2^-ε-dGuo. The human AlkB orthologues varied in their activities [139].

**Fig. 22:**
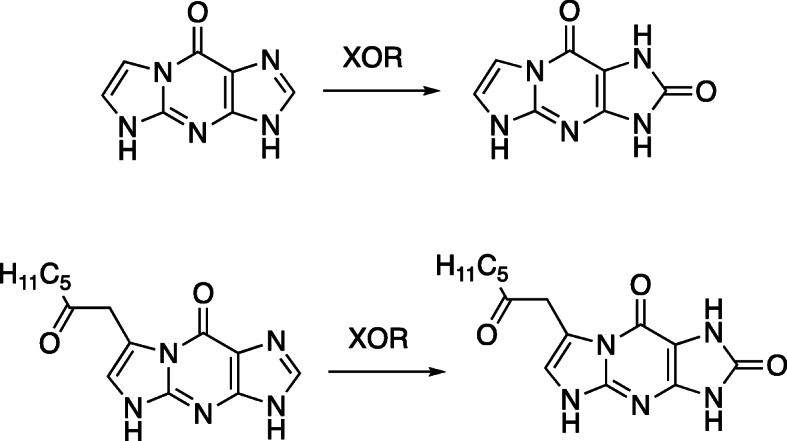
Oxidation of 1,*N*^2^-ε-Gua and heptanone-1,*N*^2^-ε-Gua by xanthine oxidoreductase (XOR) [140]

An interesting reaction occurs with 1,*N*^2^-ε-Gua and 7-(2-heptanone)-1,*N*^2^-ε-Gua, in which the 2-carbon is oxygenated by xanthine oxidoreductase (Fig. 22) [140]. No oxidation of 1,*N*^6^-ε-Ade or 3,*N*^4^-ε*-*Cyt was detected.

## Copying of 1,*N*^6^-ε-ado in DNA as well as in RNA

The incorporation of ribonucleotides during DNA replication represents a threat to the genome and its stability [141–143]. In particular, a ribo backbone may increase the risk of spontaneous hydrolysis that can lead to DNA strand breaks [144, 145], although this conclusion is controversial [146]. Embedded ribonucleotides have also been linked with systemic autoimmunity and chromosomal instability [147]. The main reasons for rNTP insertions are the higher cellular concentrations of rNTPs (over dNTPs), lack of complete sugar discrimination, and persistence of a ribo-backbone in DNA due to the incomplete removal RNA stretches from Okazaki fragments [148–151]. The human RNase H2-mediated ribonucleotide excision repair (RER) pathway helps in stabilizing genomic integrity by removing embedded ribonucleotides from DNA [152–154].

However, some ribonucleotides in DNA may persist [155], and these embedded ribonucleotides are considered a type of DNA damage. It is very important to understand the fate of ribonucleotides that escape repair. The TLS DNA damage tolerance pathway can have an important role in this context. Pol η can bypass the embedded ribonucleotides in DNA, and our own studies have shown novel functions of pol η [156–159]. However, little is known about pol η-mediated TLS across from a ribonucleotide in the DNA, and it is important to know the role of pol η in the context of ribonucleotides, either in the form of an RNA template or as ribonucleotides in DNA.

**Fig. 23:**
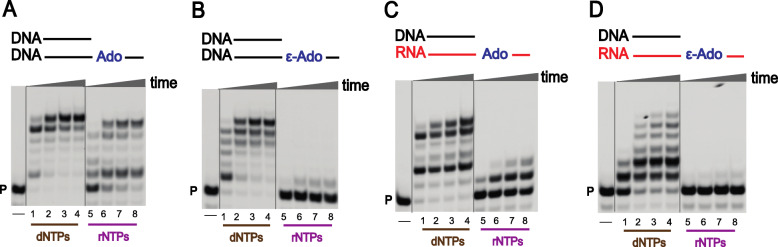
Reactions catalyzed by hpol η with 1,*N*^6^-ε-Ado. **a**, **b** full-length extension of DNA primer opposite Ado- (**a**) and ε-Ado- (**b**) containing templates using physiological concentrations of mixtures of dNTPs and rNTPs; **c**, **d** full-length extension of DNA primer opposite Ado- and ε-Ado-containing RNA templates, respectively. P indicates the 5′-FAM labeled primer, and the products of each reaction were analyzed by denaturing gel electrophoresis. Each set of lanes indicates analysis done at varying times (0, 5, 30, and 60 min). The figure is adapted from reference [159]

Our studies revealed interesting details about ribonucleotide tolerance, reverse transcription, and RNA primer extension events for the most abundant nucleotide, Ado, and its modified analog 1,*N*^6^-ε-Ado [159]. In pol η-mediated bypass studies using physiological concentrations of dNTPs, as well as rNTPs (Fig. 23), a DNA primer was fully extended using dNTPs when hybridized with an Ado-containing DNA template (DNA-Ado, Fig. 23A, lanes 1–4), but less processive extension was observed for rNTPs (Fig. 23A, lanes 5–8). pol η-mediated TLS was slower opposite 1,*N*^6^-ε-Ado as compared to Ado in a DNA template. The bypass of 1,*N*^6^-ε-Ado was inefficient using dNTPs (Fig. 23B, lanes 1–4), and with rNTPs the reactions were completely retarded (Fig. 23B, lanes 5–8). The TLS process was attenuated in the presence of 1,*N*^6^-ε-Ado (Fig. 23A, B, compare lane 1), and hpol η performed error-prone bypass of 1,*N*^6^-ε-Ado. Single nucleotide insertion and steady-state kinetic studies indicated that pol η preferably inserted dATP and dGTP opposite a 1,*N*^6^-ε-Ado-modified DNA template (compared to dTTP and dCTP). No rNTP incorporation was observed opposite 1,*N*^6^-ε-Ado in DNA, indicating that pol η follows a purine rule due to preference for adding deoxyribopurines (over ribo-purines) opposite 1,*N*^6^-ε-Ado. In steady-state kinetic analyses, dTTP insertion opposite 1,*N*^6^-ε-Ado was very unfavorable compared to other dNTPs [159].

Mass spectral analysis of pol η-mediated TLS products of the DNA/DNA-1,*N*^6^-ε-Ado complex revealed frameshifts (one deletion) opposite the lesion, using physiological concentrations of dNTPs. Thus, 1,*N*^6^-ε-Ado can exist in a staggered configuration and the polymerase may skip the lesion, and pairing of an incoming nucleotide with the next neighboring base on the template can be favored [118]. In addition to frameshifts, products with the insertion of A and G opposite 1,*N*^6^-ε-Ado lesion were also observed [159]. In addition, mis-insertion of G in the extension step was also observed [159]. Overall, steady-state kinetic and mass spectral analyses both showed the insertion of A and G opposite 1,*N*^6^-ε-Ado while mass spectral analysis revealed frameshift products opposite the lesion.

pol η-mediated reverse transcription activities were also analyzed using Ado- and 1,*N*^6^-ε-Ado-containing RNA templates (Fig. 23C-D), utilizing physiological concentrations of dNTPs as well as rNTPs. Across from the lesion, the DNA primer was extended with low processivity using a mixture of dNTPs (Fig. 23D, lanes 1–4), but extension reactions were strongly retarded with rNTPs (Fig. 23D, lanes 5–8). Thus, pol η acted as a reverse transcriptase and added only dNTPs (instead of rNTPs) opposite 1,*N*^6^-ε-Ado. The single nucleotide insertion assays showed that, for a 1,*N*^6^-ε-Ado-modified template, pol η preferably added dATP and dGTP, a similar base selectivity as observed in the TLS process. Steady-state kinetic analysis indicated that the incorporation of dTTP across 1,*N*^6^-ε-Ado was quite unfavorable, as observed in the TLS process. Overall, pol η catalyzed faithful reverse transcription opposite a 1,*N*^6^-ε-Ado-containing RNA template because of the preference for dNTPs over rNTPs [159].

RNA primer extension opposite the 1,*N*^6^-ε-Ado-containing DNA template showed that pol η-mediated RNA primer extension was severely disturbed as compared with an Ado-containing DNA template (using dNTPs). Overall, pol η follows a purine rule, with preference for dGTP insertion opposite 1,*N*^6^-ε-Ado imbedded in DNA. No rNTP incorporation was observed opposite 1,*N*^6^-ε-Ado with an RNA primer [159].

## Human RNase H2-mediated incision of 1,*N*^6^-ε-ado in DNA

**Fig. 24:**
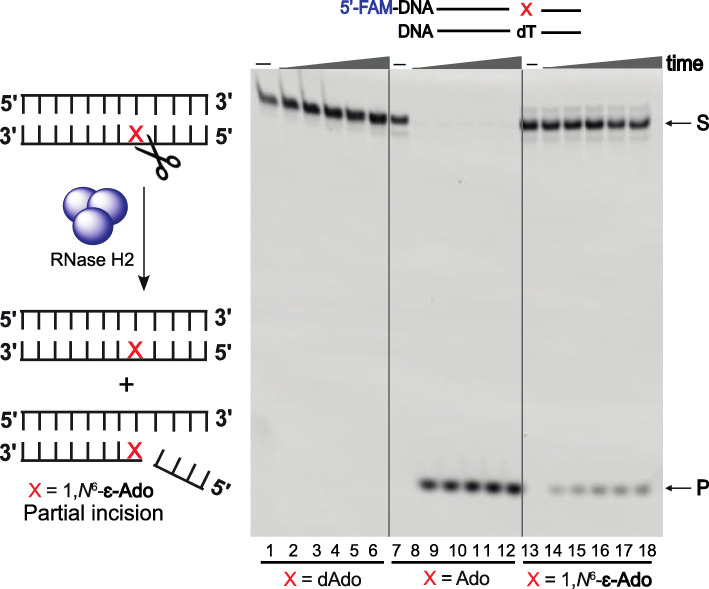
Repair of 1,*N*^6^-ε-Ado-containing DNA by RNase H2. Incision assays for DNA-X/DNA-dT employing human RNase H2. S denotes the 5′-FAM labeled substrate, and P denotes the incised product. The products of each reaction were analyzed by denaturing gel electrophoresis. Each set of lanes indicates analysis done at varying times (0, 5, 15, 30, 45, and 60 min). The figure is adapted from reference [159]

The endoribonuclease activity of human RNase H2 opposite dAdo, Ado, and 1,*N*^6^-ε-Ado in the DNA template was examined (with the complementary base T). Incision assays showed that RNase H2-mediated recognition and incision of Ado in DNA-Ado template was very efficient (Fig. 24, lanes 7–12), but the endoribonuclease activity was significantly reduced when a DNA duplex was used containing a 1,*N*^6^-ε-Ado modification (Fig. 24, lanes 13–18). These results indicate that RNase H2 is able to recognize the damaged ribonucleotide 1,*N*^6^-ε-Ado but exhibits only partial incision activity. Importantly, the human RNase H2-mediated recognition and repair of Ado and 1,*N*^6^-ε-Ado was entirely different, apparently due to the presence of the etheno group on the adenosine. If this adduct persists in the DNA and TLS is the only way of coping with 1,*N*^6^-ε-Ado, then the versatile human TLS pol η may tolerate this adduct but in an error prone way. In addition, the possibility of a base excision repair pathway in removing this adduct from DNA cannot be excluded.

## Conclusions

The etheno story is one in which chemical and biological curiosity came to have considerable relevance in biomedical applications. The story began with determination of the structures of some unusual tRNA entities, the Y-bases. The chemistry led to some useful fluorescent reagents and served as a basis for synthesis and understanding the mechanism of how these bases are formed following exposure to both exogenous and endogenous sources of *bis*-electrophiles, e.g. vinyl monomers. Relevance to the highly unusual cancers (hemangiosarcomas) related to industrial exposure to vinyl chloride was a key event, and today the most documented basis for the tumors may be the *N*^2^,3-dGuo:dTTP pairing (G to A transitions) [99, 126]. Considerable insight has been gained in the formation and repair of DNA etheno adducts. The discovery of the etheno adducts in DNA and RNA of experimental animals and humans never exposed to vinyl monomers and other prospective *bis*-electrophiles in the environment led to the discovery of the role of lipid peroxidation, and the adducts may be related to the association of cancer with different diets and lifestyles. Finally, the story has come full circle, in a sense, with the discovery that RNA etheno adducts can be copied and lead to mistakes in DNA [159].

## Data Availability

Not applicable.

## Abbreviations

Ade: Adenine
Cyt: Cytosine
Gua: Guanine
Ado: Adenosine
Cyd: Cytidine
Guo: Guanosine
dAdo: deoxyadenosine
dCyd: deoxycytidine
dGuo: deoxyguanosine
dThd: deoxythymidine
1,*N*^6^-ε-Ado: 1,*N*^6^-ethenoadenosine
3,*N*^4^-ε-Cyd: 3,*N*^4^-ethenocytidine
1,*N*^2^-ε-Gua: 1,*N*^2^-ethenoguanine
1,*N*^2^-ε-Guo: 1,*N*^2^-ethenoguanosine
*N*^2^,3-ε-Guo: 1,*N*^2^-ethenoguanosine
1,*N*^6^-ε-dAdo: 1,*N*^6^-ethenodeoxyadenosine
3,*N*^4^-ε-dCyd: 3,*N*^4^-ethenodeoxycytidine
1,*N*^2^-ε-dGuo: 1,*N*^2^-ethenodeoxyguanosine
*N*^2^,3-ε-dGuo: 1,*N*^2^-ethenodeoxyguanosine
FAM: 6-carboxyfluorescein
P450: cytochrome P450
pol: DNA polymerase
NER: Nucleotide excision repair
RER: Ribonucleotide excision repair
TLS: Translesion synthesis
